# The Chapin Home

**Published:** 1889-12

**Authors:** 


### THE CHAPIN HOME.

How quietly and with how little noise and bluster the noble work of the Chapin Home has pursued its way and kept its doors open for the reception of many, such as have not found a home elsewhere and who are not admitted to the benefits of other homes because they have not the mark of some religious organization to point to as a guarantee of their standing, as if this were the only reason for their needs or necessities. This Home is organized on entirely different principles. Rev. E. H. Chapin was formerly well known and highly honored as one of the most distinguished clergymen that ever preached in the city. After his departure to a higher sphere of existence, his noble wife, in view of the great need in many cases of the aged who, through misfortunes of various kinds, were destitute of homes where they could in their declining years be cared for and enjoy the remainder of their lives in comparative happiness, established the Home, which was incorporated in 1869, and called around her a noble band of women who, with their successors ever since, wrought faithfully to maintain the Home and make it agreeable as well as useful to those who should be so fortunate as to become inmates.

Mrs. Chapin was too much of a philanthropist to shut out many who were needy merely for the reason that they had imbibed different opinions from her, so this society proposed to take in all who were in need of such a home, without looking to their religious opinions. Consequently we now find at the Home, Episcopalians, Universalists, Presbyterians, Methodists, Dutch Reformed, Moravians, Roman Catholics, Baptists, Quakers, Spiritualists, Lutherans, Congregationalists and Liberals. It will be seen by this how well they have carried out the spirit of their organization. Not only is this the case with their religion but also nationalities, America, England, Ireland, Scotland, Denmark, France, Prussia, Canada, as their birth-places are represented.

By far the least part of the work of this truly humane society is received by Universalists. More than three-fourths of its expenses are incurred by members of other denominations. It would seem to be a matter of the strictest justice that it should be liberally supported by those societies and from those whose own members are being benefitted by its noble work. It is managed by women wholly and entirely, and is a noble example of how well they do their work and how it is managed economically and how all the inmates are content and happy in their home. It is not, like some institutions, blessed with a large endowment fund and the lady managers have each year to get up a fair to "help out" in the current expenses. This year it takes place in the Metropolitan Opera House and in the month of December, and it is to be hoped that there will be an attendance worthy of the cause.

